# Systemic Review on Transcranial Electrical Stimulation Parameters and EEG/fNIRS Features for Brain Diseases

**DOI:** 10.3389/fnins.2021.629323

**Published:** 2021-03-26

**Authors:** Dalin Yang, Yong-Il Shin, Keum-Shik Hong

**Affiliations:** ^1^School of Mechanical Engineering, Pusan National University, Busan, South Korea; ^2^Department of Rehabilitation Medicine, Pusan National University School of Medicine, Pusan National University Yangsan Hospital, Yangsan-si, South Korea

**Keywords:** transcranial electrical stimulation, transcranial direct current stimulation, transcranial alternation stimulation, transcranial random noise stimulation, electroencephalography, functional near-infrared spectroscopy, brain disease

## Abstract

**Background:**

Brain disorders are gradually becoming the leading cause of death worldwide. However, the lack of knowledge of brain disease’s underlying mechanisms and ineffective neuropharmacological therapy have led to further exploration of optimal treatments and brain monitoring techniques.

**Objective:**

This study aims to review the current state of brain disorders, which utilize transcranial electrical stimulation (tES) and daily usable noninvasive neuroimaging techniques. Furthermore, the second goal of this study is to highlight available gaps and provide a comprehensive guideline for further investigation.

**Method:**

A systematic search was conducted of the PubMed and Web of Science databases from January 2000 to October 2020 using relevant keywords. Electroencephalography (EEG) and functional near-infrared spectroscopy were selected as noninvasive neuroimaging modalities. Nine brain disorders were investigated in this study, including Alzheimer’s disease, depression, autism spectrum disorder, attention-deficit hyperactivity disorder, epilepsy, Parkinson’s disease, stroke, schizophrenia, and traumatic brain injury.

**Results:**

Sixty-seven studies (1,385 participants) were included for quantitative analysis. Most of the articles (82.6%) employed transcranial direct current stimulation as an intervention method with modulation parameters of 1 mA intensity (47.2%) for 16–20 min (69.0%) duration of stimulation in a single session (36.8%). The frontal cortex (46.4%) and the cerebral cortex (47.8%) were used as a neuroimaging modality, with the power spectrum (45.7%) commonly extracted as a quantitative EEG feature.

**Conclusion:**

An appropriate stimulation protocol applying tES as a therapy could be an effective treatment for cognitive and neurological brain disorders. However, the optimal tES criteria have not been defined; they vary across persons and disease types. Therefore, future work needs to investigate a closed-loop tES with monitoring by neuroimaging techniques to achieve personalized therapy for brain disorders.

## Introduction

Brain disorders represent a collection of syndromes characterized by abnormalities in memory, sensation, behavior, and even personality. Neurological disorders are the second leading cause of death worldwide (Feigin et al., 2019). The burden is recognized as a global public health challenge and will increase in the next few decades (Feigin et al., 2020). As reported by the World Health Organization, there are approximately 800,000 deaths from suicide each year (approximately one death every 40 s) due to mental health issues (World Health Organization, 2018). Through decades of research, health professionals have developed a set of systematic criteria for diagnosing brain disorders, together with pharmaceutical and psychological treatments; however, understanding of the neural substrates and mechanisms involved in these diseases is limited (Chou and Chouard, 2008). In addition, reliable neurological biomarkers for identifying brain disorders are insufficient (Chou and Chouard, 2008). Moreover, certain brain disorders remain substantially unaffected by neuropharmacological therapy (Ciullo et al., 2020), and the treatment options are far from optimal in terms of efficacy and specificity. Therefore, it is essential to find alternative therapies for brain disorders that are efficient in clinical practice.

**FIGURE 1:**
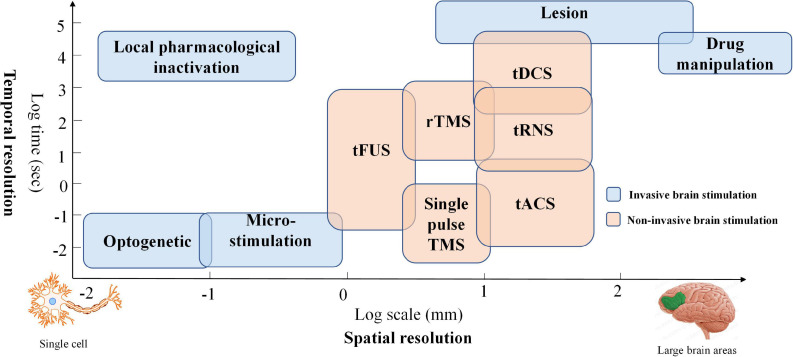
Temporal and spatial resolution of various brain intervention methods: Blue boxes represent invasive brain stimulation techniques, and orange boxes denote non-invasive stimulation methods.

Brain stimulation has been widely applied due to its ability to modulate brain plasticity in neuropsychiatric patients (Davis and Smith, 2019; Kim E. et al., 2020; Yoo et al., 2020). Typically, brain stimulation methods have been classified as invasive or noninvasive brain stimulation (NIBS). The invasive brain stimulation approach (i.e., pharmacological intervention, targeted microsimulation, optogenetics, etc.) has been commonly applied in animal models (Polanía et al., 2018). It can be used to demonstrate the relationships of its targets to brain function with high spatial precision (e.g., cell-type effect). NIBS provides a way to modulate brain function without opening the skull. Thus, many human studies have employed NIBS to explore the causal relationship between neurological function and behavior. Two mainstay NIBS approaches have emerged to treat brain disorders in the clinical context: transcranial magnetic stimulation (TMS) and transcranial electrical stimulation (tES). The principle underlying these two modalities is based on electromagnetism and harnesses weak electrical current stimulation. More specifically, TMS can depolarize neurons by generating a strong current, and tES can influence ion channels and gradients to modulate neuronal membrane potential (Fregni and Pascual-Leone, 2007). Comparing the spatial effectiveness of NIBS with other methods, TMS and tES, the best-known stimulation modalities, can generate relatively large magnetic and electrical fields in the brain. A third promising NIBS method, transcranial focused ultrasound stimulation (tFUS), provides a solution for the low degree of spatial localization in tES and TMS by projecting the acoustic intensity field beam into brain tissues (Pasquinelli et al., 2019). However, further investigation of neurophysiological foundations is required before applying tFUS as a safe therapy in daily routine (Polanía et al., 2018). Therefore, TMS and tES are the current primary noninvasive brain stimulation methods. Both modalities affect brain function by modulating the excitation or inhibition of interneuron circuits. Generally, tES can maintain a longer outlasting effect of neural excitability than can TMS (Schulz et al., 2013). In addition, tES offers the possibility of designing a reliable sham/placebo condition for double-blind controlled clinical trials since short-term tingling sensations gradually fade away after the onset of stimulation (Nitsche et al., 2008). Moreover, tES has the advantages of lower cost, portability, and ease of application. The temporal and spatial resolutions of various brain intervention methods are compared in Figure 1.

Conventionally, there are three tES modalities: transcranial direct stimulation (tDCS), transcranial alternating current stimulation (tACS), and transcranial random noise stimulation (tRNS) (Brunyé, 2018; Bikson et al., 2019). The delivery method of the current is the main difference among the three modalities. tDCS typically transfers a homogeneous direct current ranging from 1 to 2 mA from the electrodes (i.e., two or more) on the scalp to modulate brain activation (Vosskuhl et al., 2018; Yaqub et al., 2018; Brunyé et al., 2019). In the tACS case, the current is delivered in an oscillating manner with a particular frequency and stimulation amplitude. tRNS differs from tDCS and tACS. The current amplitude for tRNS is randomly applied with a normal distribution around a specific mean strength. The principle of tACS and tRNS is the modulation of ongoing neural oscillations and the induction of the neuroplastic effect by using appropriate parameter values. tDCS is used to influence neuronal excitability by membrane polarization; for example, the anode causes depolarization, and the cathode results in hyperpolarization. The effect depends on various stimulation parameters, such as polarity, stimulation duration, intensity level, and the subject’s brain state. To date, there are no clear criteria or quantitative assessment techniques to provide guidelines for tES in terms of modulation duration and intensity or the locations where electrodes should be placed.

Neuroimaging refers to the use of magnetic and other techniques to understand the living brain system, which can reflect the properties of function, structure, or change in the brain in terms of temporal (i.e., functional imaging) and spatial localization (i.e., structural and functional imaging) (Li et al., 2018; Yin et al., 2019). Neuroimaging is commonly used to diagnose neuropsychiatric disorders and evaluate effects following therapy (i.e., brain stimulation) and includes techniques such as structural magnetic resonance imaging (MRI), functional magnetic resonance imaging (fMRI), single-photon emission computed tomography (SPECT), positron emission tomography (PET), functional near-infrared spectroscopy (fNIRS), and electroencephalography (EEG) (Naseer and Hong, 2015; Hong and Khan, 2017; Hong et al., 2020). Typically, MRI, fMRI, SPECT, and PET can offer an excellent spatial resolution for brain state examinations. However, these assessments can only be performed in restricted environments due to equipment size (i.e., bulkiness or lack of mobility) (Hong and Yaqub, 2019). Moreover, some techniques (e.g., PET and SPECT) require the insertion of radioactive tracers, limiting repeated measurements, especially for children and pregnant women (Irani et al., 2007). In addition, these systems, including MRI and fMRI, are costly, highly susceptible to motion artifacts, and have a low temporal resolution (compared with EEG and fNIRS).

**FIGURE 2:**
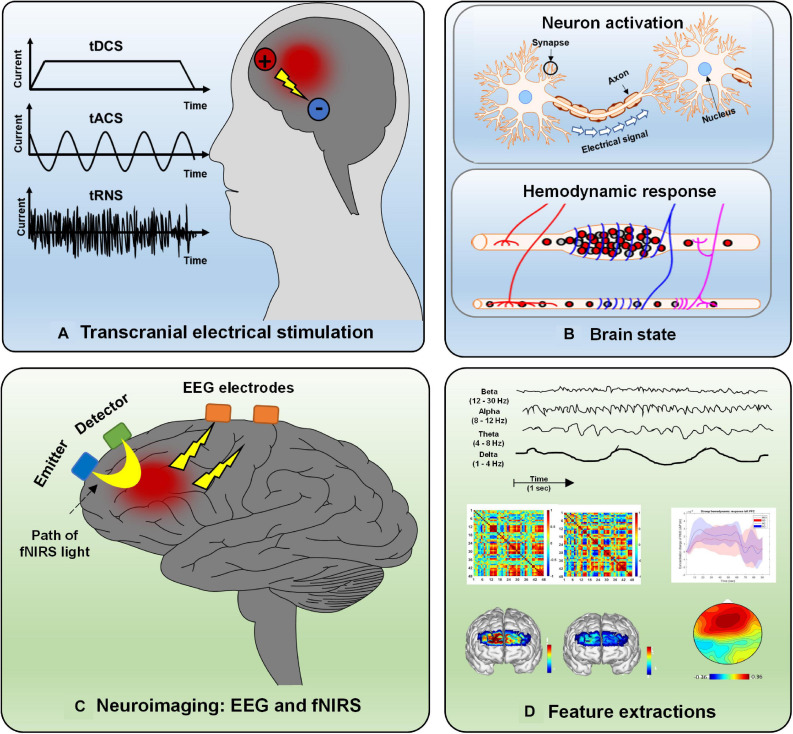
Overview of electrical stimulation, EEG, fNIRS, and feature extraction: **(A)** Three types of electrical stimulation (tDCS, tACS, and tRNS), **(B)** hemodynamic response caused by neural activity, **(C)** principle of EEG and fNIRS, and **(D)** various features extracted from neurological signals.

As a noninvasive neuroimaging modality, EEG is one of the oldest techniques used to measure neural activation in the human brain for diagnosis or brain–computer interface (BCI) purposes (Naseer and Hong, 2013; Khan and Hong, 2017; Tanveer et al., 2019). Since they are portable and have the advantage of higher temporal resolution, EEG-based BCI applications have been widely designed for daily use (e.g., home automation control devices, EEG-based wheelchairs, brain disorder detection platforms) (Kim et al., 2019; Lee T. et al., 2020; Rashid et al., 2020; Yang et al., 2020b). EEG measurements are based on electrical potential differences between different electrodes on the scalp. A potential difference is caused by the propagation of the current flow induced by synchronized postsynaptic potentials in pyramidal neuron cell membranes. fNIRS is a promising noninvasive neuroimaging technique featuring the advantages of safety, low cost, mobility, excellent temporal resolution (compared with fMRI), moderate spatial resolution, and tolerance to motion artifacts (Nguyen et al., 2016; Ghafoor et al., 2019). The fNIRS principle is based on the absorption characteristics of oxygenated hemoglobin (HbO) and deoxygenated hemoglobin (HbR) in the spectrum ranging from 650 to 1,000 nm, for which brain tissues are more translucent than HbO or HbR (Aqil et al., 2012a,b). Changes in blood flow (i.e., increases or decreases) reflect a local brain region’s hemodynamic activity resulting from neuronal firing. More specifically, activation of brain cortical neurons results in greater blood flow (detected by the surplus of oxyhemoglobin in veins) than in brain regions with inactive neurons (Berger et al., 2019; Hu et al., 2019; Shin, 2020). Types of tES, the hemodynamic response caused by neural activity, EEG and fNIRS principles, and examples of extracted features are depicted in Figure 2. EEG and fNIRS are widely applied in clinical brain state monitoring (Yang et al., 2019b, 2020a). The development of therapeutic strategies for neuropsychiatric disorders is based on the properties described above: minimal invasiveness, safety, ease of use, and repeatability (Dubreuil-Vall et al., 2019; Ermolova et al., 2019).

This study had two goals. The first aim was to review the current state of the application of tES, monitored by EEG and fNIRS, for treatment of nine common brain disorders: Alzheimer’s disease (AD) and mild cognitive impairment (MCI), depression, autism spectrum disorder (ASD), attention-deficit hyperactivity disorder (ADHD), epilepsy, schizophrenia, Parkinson’s disease (PD), stroke, and traumatic brain injury (TBI). Second, this study aimed to provide a general reference for future investigation of tES as a treatment for the nine diseases mentioned above: (i) how to conduct the stimulation (i.e., duration and intensity), (ii) where the electrodes should be placed, (iii) which types of EEG and fNIRS features can be used to evaluate the stimulation effect, and (iv) the behavioral and neurological effects following stimulation.

## Methods

**FIGURE 3:**
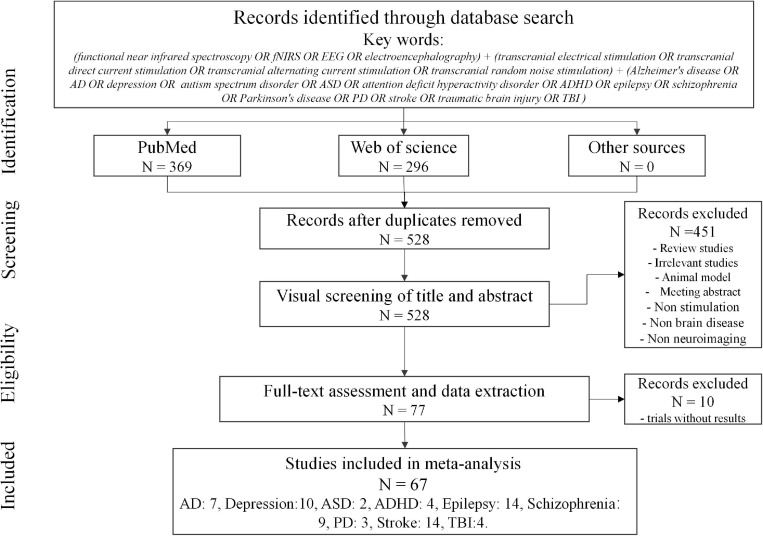
Overview of literature search for review.

In this study, a two-stage literature search was performed to identify relevant investigations. First, an online search was conducted using the PubMed and Web of Science databases, which included peer-reviewed articles from January 2000 to October 2020 with the following keywords: (functional near-infrared spectroscopy OR fNIRS OR EEG OR electroencephalography) ++ (transcranial electrical stimulation OR transcranial direct current stimulation OR transcranial alternating current stimulation OR transcranial random noise stimulation) + Alzheimer’s disease OR AD OR depression OR autism spectrum disorder OR ASD OR attention-deficit hyperactivity disorder OR ADHD OR epilepsy OR schizophrenia OR Parkinson’s disease OR PD OR stroke OR traumatic brain injury OR TBI). Second, an additional literature search was conducted through the reference lists in selected studies or the related review paper. The aim of this two-stage literature search was to ensure that the studies included were as comprehensive as possible. As shown in Figure 3, the search identified 665 studies (i.e., 369 from PubMed and 296 from Web of Science). After removing 137 duplicates and 451 studies that did not relate to this review (i.e., review studies, animal model studies, meeting abstracts, investigations lacking stimulation or not involving brain disease, and those lacking EEG or fNIRS), 77 articles remained. Since several studies did not show the results (i.e., 10 trial studies), this systematized review consisted of 67 data sets (i.e., AD, 7; depression, 10; ASD, 2; ADHD, 4; epilepsy, 14; schizophrenia, 9; PD, 3; stroke, 14; and TBI, 4).

## Alzheimer’s Diseases and Mild Cognitive Impairment

Approximately 60–70% of dementia is caused by AD. AD is a progressive brain disorder in which memory and cognitive function cause increasing impairment until death (Vuksanović et al., 2019; Feng et al., 2020). To date, there is no pharmacological cure available, except for treatment to manage symptoms. MCI is considered to be the middle stage between healthy controls and patients with dementia. It has been reported that approximately 32–38% of patients with MCI develop dementia within five or more years (Alzheimer’s Association, 2020). Brain stimulation is a promising therapeutic approach for improving memory and cognitive function in AD and preventing the progression from MCI to AD.

As shown in Table 1, six studies addressed AD (four studies) and MCI (three studies), and one was related to frontotemporal dementia. EEG neuroimaging techniques were conducted in all studies. The features extracted from EEG included the event-related potential, coherence, connectivity, and power spectra in different brain bands (e.g., alpha, beta, theta, gamma, etc.). The stimulation duration and intensity also differed for each study. One of the tACS studies (Naro et al., 2016) applied tACS to modulate gamma-band oscillations (GBOs) in AD, MCI, and HC groups. Anodal electrodes were positioned on the primary motor area (M1), premotor area (PMA), supplementary motor area (SMA), dorsolateral prefrontal cortex (DLPFC), and dorsomedial prefrontal cortex (DMPFC). The reference electrode was placed on the right mastoid. The entire stimulation procedure lasted 10 min with a 40–120-Hz stimulation frequency and a 1-mA current intensity. The tACS (GBO and neuropsychological test) effect differed among the three groups (AD, MCI, and HC). In the HC group, GBO increased. Partial improvement of GBO was observed in the MCI group, whereas there was no significant effect for individuals with AD. Interestingly, after 2 years of follow-up, the individuals with MCI who failed to show a significant effect had progressed to AD. These results indicate the difficulty of employing tACS to modulate the neuroplasticity of patients with AD using the stimulation parameters mentioned above. In another slow-oscillation tDCS (so-tDCS) study (Ladenbauer et al., 2017), the authors used slow oscillatory stimulation at 0.75 Hz to modulate the brain activity pattern and memory consolidation during the daytime nap period in patients with MCI, with the anodes and cathodes placed on the frontal area (F3 and F4) and mastoid location, respectively. The slow cortical oscillation (0.5–1 Hz), spindle power, and declarative memory significantly improved after stimulation compared to the sham group. Similarly, the authors of one of the frontotemporal dementia studies (Ferrucci et al., 2018) stated that the effect of anodal tDCS (current, 2 mA; duration, 20 min; anode, frontotemporal cortex; cathode, right deltoid muscle) might be correlated with low-frequency band oscillation during the attentional processes. Two of the AD studies were quite similar. Both studies used tDCS with a 1.5-mA current to conduct the stimulation within a day visit. The most significant differences were in the duration and location of the simulation. The first study (Marceglia et al., 2016) delivered 15 min of stimulation from the bilateral temporoparietal area (left side, P3-T5; right side, P6-T4) with the reference on the right deltoid muscle. The results revealed that high-frequency power in the temporoparietal area and coherence at the temporal–parietal–occipital junction were increased in the AD group. Stimulation was applied in the second study (Cespón et al., 2019) over the left DLPFC, and the return electrode was placed on the right shoulder, with a stimulation duration of 13 min. The working memory, P200 amplitude, and theta band brain activity in the frontal area increased after cathodal tDCS. These two studies indicate that brain stimulation may rely significantly on the interaction among tDCS polarity, stimulation location, and brain state. One of the MCI studies (Emonson et al., 2019) applied an extended stimulation period (i.e., 20 min) to deliver the stimulation current (1 mA) from the left DLPFC (F3) to the contralateral supraorbital area (Fp2). Event-related potential (ERP) differed before and after stimulation for younger and older adult groups. However, this phenomenon was not observed in the MCI group. This finding is consistent with that of prior AD studies (Cespón et al., 2019). To compare the effects of short- and long-term tDCS, Gangemi et al. (2020) suggested that if brain stimulation (i.e., tDCS) can be effective with steady-state neurocognitive function over the short term, then this effect could be prolonged for 8 months. In addition, this study (current, 2 mA; duration, 20 min; visit, 10 days or 8 months; anode, left frontotemporal cortex; cathode, right frontal lobe) (Gangemi et al., 2020) demonstrated that anodal tDCS provides a technique to slow the progression of AD by influencing neurological patterns in the brain.

**TABLE 1:** Studies and experimental characteristics of tES literature for AD and MCI.

**Authors**	**Subjects**	**Stimulation parameters**				**Neuroimaging**		**Conclusion**
		**Type**	**Current**	**Duration**	**Location**	**Type**	**Feature**	
Gangemi et al., 2020	26	tDCS	2 mA, 20 min	Daily for 10 sessions/80 sessions for 8 months	Anode: left frontotemporal (F7-T3), cathode: right frontal lobe (Fp2).	EEG	Alpha/Beta/Theta rhythm	The short- and long-term anodal-tDCS can be used as an effective treatment to slow the progression of dementia.
Emonson et al., 2019	49	tDCS	1 mA, 20 min	1 session	Anode: LDPFC (F3), cathode: contralateral supraorbital area (Fp2)	EEG	Event-related protentional	The manifestation and nature of tDCS induced neurobiological effects to differ based on age and the presence or absence of cognitive impairment.
Ferrucci et al., 2018	13	tDCS	2 mA, 20 min	Daily for 5 consecutive days	Anode: frontal-temporal lobes bilaterally(F7-F8), cathode: Right deltoid muscle	EEG	Power spectrum (2–7 Hz) and high (8–25 Hz) frequency	Anodal tDCS applied over the bilateral frontal-temporal cortex significantly improves cognitive ability.
Ladenbauer et al., 2017	16	So-tDCS	0.75 Hz, 0.522 mA/cm^2^, 5 min	1 session (3–5 blocks)	Anode: prefrontal cortex (F3-F4), cathode: ipsilateral mastoid	EEG	Power spectrum (0.5–1 Hz) and fast spindles (12–15 Hz), phase-amplitude coupling	A well-tolerated therapeutic approach for disordered sleep physiology and memory deficits in patients with MCI and advances our understanding of offline memory consolidation.
Marceglia et al., 2016	7	tDCS	1.5 mA, 15 min	1 session	Anode: bilateral temporal-parietal area, cathode: right deltoid muscle.	EEG	Power spectral in low (2–7 Hz) and high (8–25 Hz) frequency, coherences	The modulation of cortical activity supports anodal tDCS benefits in patients with patients during working memory tasks.
Cespón et al., 2019	26	tDCS	1.5 mA, 13 min	1 session	Anode: left DLPFC (F3), cathode: right shoulder. Anode: M1 (C3), DLPFC	EEG	ERP, power spectrum in theta (4.1–7.9 Hz), alpha (8.1–13.9 Hz) and beta (15.1–24.6 Hz)	Functional neural modulations were promoted by anodal tDCS in healthy elderly and by cathodal tDCS in patients with AD
Naro et al., 2016	87	tACS	1 mA, 40–120 Hz, 10 min	31 session	(AF3-AF7), DMPFC (AF3-F1), PMA (FC3), or SMA (FCz) of the left hemisphere, cathode: right mastoid	EEG	Power spectrum in the gamma band	tACS can provide a novel way to diagnose MCI and AD, and it can identify patients with MCI at risk of developing dementia

## Depressive Disorder

Depression presents with sorrow, guilt, fatigue, low self-worth, irregular sleep/appetite, loss of interest, and low concentration (Xu J. et al., 2019; Olejarczyk et al., 2020). Commonly, these symptoms persist in the long term and recur easily. It substantially influences the patient’s daily life. In the severe stage, depression may result in suicide. This disorder also occurs in children and adolescents (under 15 years old), but this age group’s prevalence is lower than in adults. Researchers have found altered electrical activity in specific brain regions in patients with depression with neural science development. The cortex represents excitability, and other regions exhibit lower activities. tES, as an electrical stimulation therapy modality, has shown a promising advantage for improving the symptoms of depression.

Ten studies of depression were included in this review article, summarized in Table 2. Nine papers used EEG as the neuroimaging method to evaluate the neurological effect, and only one fNIRS study assessed therapeutic performance from a hemodynamic perspective. All EEG studies employed the alpha band power spectrum to compare the difference between the pre- and poststimulation. Some studies have considered an alpha band from 8 to 12 Hz, while others regard the ranges between 8 and 13 Hz or 7.5 and 12 Hz as the alpha band. The ERP power spectra in the delta, theta, beta, and gamma bands were also investigated as extracted EEG-based features. In addition to the standard features extracted from the alpha band, all anode stimulation locations for tACS and tDCS included the left DLPFC (F3). A possible explanation is that the left DLPFC is associated with right prefrontal hyperfunction and dysfunction of brain plasticity in depression.

**TABLE 2:** Studies and experimental characteristics of the tES literature for depression.

**Authors**	**Subjects**	**Type**	**Stimulation parameters**			**Neuroim aging**		**Conclusion**
			**Current**	**Duration**	**Location**	**Type**	**Feature**	
Nikolin et al., 2020	20	tDCS	2 mA, 40 min	3 sessions per week for 6 weeks	Anode: left DLPFC (F3), cathode: right shoulder.	EEG	Power spectral in alpha (8–13 Hz) and theta (4–8 Hz), Event-related potential.	There is a significant improvement on the behavioral performance (i.e., mood, memory, cognitive).
Nishida et al., 2019	33	tDCS	1 mA, 20 min	1 session	Anode: left DLPFC (F5) or DMPFC (Afz), cathode: left shoulder.	EEG	Power spectral in alpha (8–12 Hz) and theta band (4–8 Hz), event-related potential.	tDCS could affect the brain activity on the stimulated brain area and influence the other related resting state neural network’s cortical brain state.
Li et al., 2019	26	tDCS	2 mA, 20 min	5 sessions/week for 4 weeks	Anode: left DLPFC (F3), cathode: right DLPFC (F4).	fNIR S	Concentration change in HbO	tDCS can improve depression symptoms in behavioral domains) and influence hemodynamic metabolism.
Alexander et al., 2019	32	tACS	1/2 mA, 10/40 Hz, 40 min	5 sessions in consecutive days	Anode: left/right DLPFC (F3/F4), cathode: Cz.	EEG	Power spectral in alpha (8–12 Hz).	10-Hz tACS could significantly reduce the alpha power over the left frontal cortex. tACS has potential for the treatment of depression.
Shahsavar et al., 2018	7	tDCS	1 mA, 20 min	5 session in consecutive days	Anode: left DLPFC (F3), cathode: right DLPFC (F4).	EEG	Event-related potential and the power spectrum in the different brain wave band	It was possible to estimate the change of depressed patients treated with tDCS with reasonable precision using the alpha band wavelet coefficients.
Al-Kaysi et al., 2017	10	tDCS	2 mA, 20 min	5 sessions per week for 3 weeks	Anode: left DLPFC (F3), cathode: F8	EEG	Power spectral in theta band (4–8 Hz), alpha (8–12 Hz), beta (13–30 Hz), and, gamma (30–100 Hz).	This study demonstrated the feasibility of predicting tDCS treatment outcomes by analyzing the EEG data recorded at baseline.
Liu et al., 2016	37	tDCS	2 mA, 20 min	5 session in consecutive days	Anode: left DLPFC (F3), cathode: right supraorbital area	EEG	Power spectral in alpha (8–13 Hz) and theta band (4–8 Hz).	tDCS could improve the depression symptom, but memory function was not immediately following or persisting after the stimulation
Powell et al., 2014	18	tDCS	2 mA, 20 min	1 session	Anode: left DLPFC (F3), cathode: F8	EEG	Power spectral in alpha (8–12 Hz) and theta band (4–8 Hz), event-related potential.	Anodal tDCS with a single session from the left DLPFC for the major depressive episode resulted in modulated brain activity of EEG.
Palm et al., 2009	1	tDCS	1 mA, 20 min	16 sessions in 27 days	Anode: F left DLPFC (F3), cathode: right supraorbital area	EEG	Power spectral in delta (1–3 Hz), theta band (4–7 Hz), alpha (8–12 Hz), and beta (13—5 Hz).	tDCS did not exert clinically meaningful antidepressant effects. The results for cognitive measures and EEG suggest that beneficial effects may occur in depressed subjects.
Khayyer et al., 2018	9	tDCS	1.5 mA, 15 min	3 sessions per week for 4 weeks	Anode: left or right DLPFC (F3 or F4), cathode: Cz.	EEG	Power spectrum in Delta (1–4 Hz), Theta (4.5–7 Hz), Alpha (7.5–12 Hz), Beta (12.5–24.5 Hz), High Beta (25–30 Hz)	The combined treatment of positive psychotherapy and tDCS showed the great performance to improve the neurological and clinical condition of major depressive disorder.

In the fNIRS study (Li et al., 2019), 26 patients with poststroke depression received tDCS for 20 min with a 2-mA current on the left DLPFC and a return electrode at the right DLPFC. Each subject underwent five stimulation sessions weekly for 4 weeks. The results showed that working memory task performance and HbO concentration were improved after the treatment. Alexander et al. (2019) examined the feasibility and efficacy of modulating alpha oscillations by applying tACS at 10 and 40 Hz in the DLPFC (left DLPFC, F3; right DLPFC, F4) (Alexander et al., 2019). For 5 consecutive days, subjects received 40 min of either 10 Hz tACS, 40 Hz tACS, or sham trial. Reduced power in the alpha band was observed after 2 weeks of the intervention applying the 10-Hz tACS protocol.

Similarly, one tDCS study (Nikolin et al., 2020) also used a 2-mA stimulation current for 40 min. The current was delivered from the left DLPFC to the right shoulder, with patients receiving the therapy three times per week for 6 weeks. Although behavioral performance (e.g., mood and working memory) improved, neurophysiological measurements (e.g., power spectrum and ERP) did not significantly change following the intervention. One comparative study (Nishida et al., 2019) conducted tDCS at an intensity of 1 mA for 20 min from the left DLPFC and DMPFC. The return electrode at the left shoulder indicated that the effect of tDCS on anxiety reduction depends on the site of stimulation. The effect of tDCS stimulation at the left DLPFC on patients with depression was correlated with theta band activity acquired from the rostral anterior cingulate cortex. The effect of DMPFC stimulation on anxiety reduction was related to alpha-band activity in the left inferior parietal lobule. Three investigations were performed applying current from the left DLPFC to the right supraorbital area with a current intensity of 2 mA for 20 min. These studies differed in duration of stimulation periods: 1 day, 5 consecutive days, and 5 consecutive days each week for 3 weeks. In another study (Powell et al., 2014), a significant reduction in the N200 amplitude and theta band activity was observed over the memory task’s frontal cortex substrate with one stimulation period. After five tDCS sessions, the depression score and the power spectrum (delta, theta, and low alpha band) were improved compared to the sham group, but this improvement was not sustained for 4 weeks (Liu et al., 2016). Long-term tDCS with 15 treatment periods has been found to affect mood and cognition in 50 and 60%, respectively, of individuals with depression (Al-Kaysi et al., 2017). Similarly, a lower-intensity (1 and 1.5 mA) tDCS protocol also resulted in reduced ERP (Shahsavar et al., 2018) and power spectra (e.g., delta, theta, and alpha frequency bands) (Palm et al., 2009; Khayyer et al., 2018) following stimulation. In particular, combined treatment with positive psychotherapy and tDCS can significantly affect depression. Effects are more pronounced following 3 months of follow-up than with the use of tDCS alone (Khayyer et al., 2018).

## Autism Spectrum Disorder

ASD refers to a developmental disorder characterized by (i) impaired communication, social behavior, language expression, and (ii) repetition of limited interests and activities. Typically, it begins in childhood and persists in adolescence and adulthood. Most individuals with ASD cannot live independently and require life-long care (Górriz et al., 2019). Several psychosocial interventions (behavioral treatment and skill training) have been applied to reduce communication difficulties and improve individuals’ quality of life with ASD. However, the effect of psychosocial treatment depends on the individual’s state. In addition to behavioral treatment, brain stimulation, as an alternative strategy, may improve symptoms from a neurological perspective by modulating deficient neural patterns.

The presence of abnormal gamma oscillations has been considered a biomarker and a target for therapeutic engagement; thus, tACS with a frequency-specific paradigm in the gamma band became the most suitable stimulation modality (Kayarian et al., 2020). Gamma band stimulation may also play a role in mediating the motor learning mechanism. Application of tACS at 70 Hz increased motor learning capacity compared to sham treatment and tACS at 10 and 20 Hz (Sugata et al., 2018). For exploring multiple therapeutic strategies for patients with ASD, tDCS was used to modulate brain function in patients from different perspectives (abnormal synaptic maturation and connectivity). Two studies employed a 1-mA current intensity for 20 min to stimulate ASD models at the DLPFC region and evaluated EEG output effects. One study (Amatachaya et al., 2015) used one tDCS treatment period. The poststimulation results showed significant improvement in the autism treatment evaluation checklist in the social and behavioral domains compared to prestimulation scores. Besides, peak values of the alpha band also increased after stimulation. Similarly, Kang et al. (2018) conducted tDCS treatments every 2 days for 10 sessions. The maximum entropy ratio (an index of EEG signal complexity) was extracted to examine the tDCS effect and increase significantly following treatment. tDCS may be capable of rehabilitating children with ASD, as might tACS with frequency-specific characteristics. The related ASD studies are listed in Table 3.

**TABLE 3:** Studies and experimental characteristics of tES literature for ASD.

**Authors**	**Subjects**	**Stimulation parameters**				**Neuroimaging**		**Conclusion**
		**Type**	**Current**	**Duration**	**Location**	**Type**	**Feature**	
Kang et al., 2018	13	tDCS	1 mA, 20 min	10 sessions	Anode: DLPFC, cathode: right supraorbital	EEG	Complexity	The complexity of EEG significantly increased after tDCS. This study suggests that tDCS may be a helpful tool for the rehabilitation of children with ASD.
Amatachaya et al., 2015	24	tDCS	1 mA, 20 min	1 session	Anode: left DLPFC (F3), cathode: right shoulder	EEG	Peak alpha frequency	The clinical (autism treatment evaluation checklist) and neurological (peak alpha frequency) performance was improved after the treatment of active tDCS.

## Attention-Deficit Hyperactivity Disorder

ADHD is also a developmental disorder characterized by two types of symptoms: (i) inattentiveness and (ii) hyperactivity/impulsiveness. Most cases are diagnosed at the ages of 6–12 years. Symptoms become particularly noticeable when circumstances change. Moreover, ADHD is commonly comorbid with other psychiatric disorders (e.g., depression and anxiety disorder), causing a substantial burden for patients and their families. Thus far, the medication-based intervention can achieve short-term effects, and the long-term effects of treatment for ADHD remain uncertain (Posner et al., 2020). It is essential to develop novel alternative strategies for treating ADHD.

There are four ADHD-related articles listed in Table 4, and all the studies used EEG for neuroimaging. Event-related potentials (P200 and P300) were employed in two of the papers and were used to evaluate the effects of tDCS (Breitling et al., 2020) and tACS (Dallmer-Zerbe et al., 2020), respectively. The remaining studies extracted functional brain connectivity (Cosmo et al., 2015b), power spectra (Dallmer-Zerbe et al., 2020), and statistical analysis (Cosmo et al., 2015a) as features for examining the neurological changes following tDCS. In all investigations, 20 min of tES therapy at low current intensity (1 mA) was applied. In the tACS study (Dallmer-Zerbe et al., 2020), the authors applied a stimulation with a mean frequency of 3 Hz, delivered from multiple electrodes (anodes: C3, C4, CP3, CP4, P3, and P4) and returned by cathodes at T7, T8, TP7, TP8, P7, and P8 (the distribution of electrodes following the international 10–20 EEG system). The neurological results following tACS demonstrated that the P300 amplitude significantly increased, accompanied by a decrease in omission errors compared to pre-tACS. Both tDCS studies share the same experimental paradigm (current intensity, duration, and stimulation location), but the results were reversed. The first study (Cosmo et al., 2015b) indicated that resting-state brain connectivity increased in individuals after DLPFC stimulation. The authors of the second study (Cosmo et al., 2015a) found no evidence supporting the capability of tDCS to improve inhibitory control by stimulating the left DLPFC in patients with ADHD performing the go/no–go task. A recent investigation (Breitling et al., 2020) compared the effectiveness of conventional (with one anodal electrode) and high-definition tDCS (with four anodal electrodes) for improving working memory performance, with the anode located near the right inferior frontal gyrus and the cathode placed over the contralateral supraorbital region. The results for working memory behavior were not generally influenced by conventional and high-definition tDCS (HD-tDCS). However, elevated P300 and N200 were observed after conventional and HD-tDCS since the current intensity differed between conventional tDCS (1 mA) and HD-tDCS (0.5 mA). The conclusion, which may be difficult to accept, is that HD-tDCS is equally suitable as conventional tDCS for improving the working memory performance of patients with ADHD. Therefore, comprehensive investigations are required to assess the effectiveness of tES for treating ADHD in the future.

**TABLE 4:** Studies and experimental characteristics of tES literature for ADHD.

**Authors**	**Subjects**	**Stimulation parameters**				**Neuroimaging**		**Conclusion**
		**Type**	**Current**	**Duration**	**Location**	**Type**	**Feature**	
Breitling et al., 2020	15	tDCS/HD- tDCS	1 mA/0.5 mA, 20 min	1 session	Anode: right IFG (F8), cathode: contralateral supra-orbital	EEG	N-200 and P300	HD-tDCS could be equally suitable with typical tDCS for improving the working memory processing of patients with ADHD.
Dallmer-Zerbe et al., 2020	18	tACS	1 mA, 20 min, 3 Hz	1 session	Anode: motor-parietal cortex (C3, C4, CP3, Cp4, P3, P4); Cathode: temporal-parietal (T7, T8, TP7, TP8, P7, P8)	EEG	P300	A significant increase in P300 amplitude in the stimulation group, which was accompanied by a decrease in omission errors pre-to-post tACS.
Cosmo et al., 2015b	60	tDCS	1 mA, 20 min	1 session	Anode: left DLPFC (F3), Cathode: right DLPFC (F4).	EEG	Functional cortical network	Anodal tDCS increased the functional brain connectivity in individuals with ADHD compared to data recorded in the baseline resting state.
Cosmo et al., 2015a	60	tDCS	1 mA, 20 min	1 session	Anode: left DLPFC (F3), cathode: right DLPF)^p z(F4).	EEG	Statistical analysis	The statistical analysis indicated that anodal stimulation over the LDPFC could not improve inhibitory control in patients with ADHD.

## Epilepsy

Epilepsy is a chronic brain disease characterized by brief involuntary movement in part or the entire body with recurrent unprovoked seizures. Sometimes, seizures result in loss of consciousness and control of bladder function. Patients with epilepsy have three times the risk of premature death as the general population. Fortunately, approximately 70% of epilepsy cases can be controlled using proper antiseizure medication. It is suggested that in the remaining patients (approximately 30%) with drug-resistant epilepsy, seizure control should be achievable through surgery and neurostimulation therapies (Devinsky et al., 2018).

Abnormal EEG patterns are among the most consistent predictors of seizure recurrence (Scheffer et al., 2009). As shown in Table 5, EEG was employed in all 14 epilepsy studies as the neuroimaging method to assess the tES effect. The evaluated features included the power spectrum, connectivity, mean peak amplitude, mean number of spikers, and seizure frequency. Each study applied a different stimulation strategy to explore the optimal stimulation protocol. In the tACS study (San-Juan et al., 2016), patients received a sinusoidal fluctuating current (frequency, 3 Hz; intensity, 1 mA) from Fp1 to Fp2. The stimulated location was determined by visual inspection of EEG signals for the most active epileptiform cortex. Sessions of 60-min duration were conducted daily for 4 consecutive days. At the 2-month follow-up, patients were asked to report whether they had experienced one or more seizures in the previous 15 days. A lower oscillation (0.75 Hz) tACS was used to investigate the enhancement of memory consolidation during slow-wave sleep in patients with temporal lobe epilepsy (TLE) (Del Felice et al., 2015). The anode was placed over the frontotemporal lobe, and the cathode was placed on the ipsilateral mastoid. Visuospatial memory performance, slow spindles (10–12 Hz), and fast spindles (12–14 Hz) were extracted as EEG features to assess the effect of stimulation. Both behavioral and neurological performance improved following stimulation. The results suggest that memory rehabilitation may be achieved by slow oscillatory stimulation in patients with TLE.

**TABLE 5:** Studies and experimental characteristics of tES literature for epilepsy.

**Authors**	**Subjects**	**Stimulation parameters**				**Neuroimaging**		**Conclusion**
		**Type**	**Current**	**Duration**	**Location**	**Type**	**Feature**	
Meiron et al., 2019	1	HD-tDCS	1 mA, 20 min	5 sessions per week for 4 weeks	Anode: frontal-parietal cortex (AF8, F2, C2, PO4), cathode :C6	EEG	Power spectral in theta band (4–8 Hz), alpha (8–12 Hz), beta (13–30 Hz), spike frequency, duration, and amplitude.	tDCS reduces the interictal epileptic discharges and change in seizure-related delta activity.
Yang et al., 2019a	7	tDCS	1 or 2 mA, 40 min	14 sessions consecutive days	Anode: left or right supra-orbital area, cathode: P4 or P3	EEG	Seizure frequency and seizure reduction.	Repeated tDCS (cathode located in the bilateral parietal area) could safely reduce seizure frequency for epilepsy patients.
Lin et al., 2018	9	tDCS	2 mA, 20 min	6 sessions in one month	Anode: contralateral shoulder area, cathode: epileptogenic focus	EEG	Seizure frequency and phase lag index/	tDCS may be considered an alternative treatment option for patients with refractory epilepsy. Its effect might be cumulative after repeated stimulations and associated with a decrease in the phase lag index.
Tecchio et al, 2018	6	tDCS	1 mA, 20 min	1 session	Anode: opposite homologous, cathode: epileptogenic focus.	EEG	Functional connectivity and power spectrum.	The neurological alternation (functional connectivity) indicated that the cathode tDCS might contribute to epilepsy and provide a new therapy to modulate the epileptic people.
Meiron et al., 2018	1	HD-tDCS	0.1–1 mA, 20 min	5 sessions per week for 2 weeks	Anode: PO3, P6, AF3, F6, FC4, O1, CP3, C1, FC8, C6, FCz, FC3, O4, F2, CP4, PO4, O2, AF8, C2, cathode: C2, TP8, CP8, O3, TP8, T8	EEG	Mean number spikers, mean peak amplitude, mean absolute power.	HD-tDCS showed safety and feasibility of early-onset epileptic encephalopathy. It provides the first evidence of HD-tDCS effects on paroxysmal EEG features in electroclinical cases under the age of 36 months. Extending HD-tDCS treatment may enhance electrographic findings and clinical effects.
Karvigh et al., 2017	10	HD-tDCS	2 mA, 20 min	10 consecutive days	Anode: frontal-parietal-temporal cortex (F3/F4, P3/P4, Cz, T3/T4) Cathode: PF1/PF2, Fz, Tz/T8, C3/C4	EEG	Seizure frequency	The statistical analysis for the whole group does not show the effect of the tDCS since the change of epileptiform discharge was not significant. However, the clinical score (i.e., working memory performance) was improved.
San-Juan et al., 2016	1	tACS	1 mA, 3 Hz, 60 min	4 sessions consecutive days	Anode: frontal cortex (Fp1 and Fp2)	EEG	Spike-low wave at 3 Hz, polis piker-slow wave at 3–4 Hz, and slow rhythmic waves at 4 Hz	At the 1-month follow-up, the patients reported a 75% increase in seizure frequency. At the 2-month follow-up, the patient reported a 15-day seizure-free period.
Tekturk et al., 2016	12	tDCS	2 mA, 30 min	3 sessions consecutive days	Anode: temporal region (T3 and T4); cathode: contralateral supraorbital region	EEG	Seizure frequency	Our small series suggested that cathodal tDCS may be used as an additional treatment option in MTLE-HS. It may be tried in patients with TLE-HS waiting for or rejecting epilepsy surgery or even with ineffective surgical results.
Liu et al., 2016	37	tDCS	2 mA, 20 mins	5 sessions Consecutive days	Anode: DLPFC (F3, F4), cathode: right supraorbital area	EEG	Power spectral in delta (1–4 Hz), theta band (5–7 Hz), low alpha (8–10 Hz), high alpha (11–13 Hz), beta (14–32 Hz), low gamma (33–35 Hz).	tDCS improved the symptoms of depression for temporal lobe epilepsy. There were no changes in memory function immediately following or persisting after a stimulation course.
Del Felice et al., 2015	12	So-tDCS	0.75 Hz, 30 mins	1 session	Anode: frontal-temporal (F7-T3 or F8-T8), cathode: ipsilateral mastoid	EEG	Spindle frequency and Cortical sources	Anodal so-tDCS over the affected temporal lobe improves declarative and visuospatial memoryperformance by modulating slow sleep spindles cortical source generators.
Auvichayapat et al., 2013	36	tDCS	1 mA, 20 min	1 session	Anode: Contralateral shoulder area, cathode: epileptogenic focus	EEG	Spikes and sharp waves	A single session of cathodal tDCS improves epileptic EEG abnormalities for 48 h and is well tolerated in children.
Fregni et al., 2006	19	tDCS	1 mA, 20 min	1 session	Anode: silent area. cathode: epileptogenic focus	EEG	Seizure frequency	Cathodal tDCS polarization does not induce seizures and is well tolerated in patients with refractory epilepsy and MCDs. tDCS might have an antiepileptic effect based on clinical and electrophysiological criteria.
Faria et al., 2012	17	tDCS	1 mA, 30 min	3 sections for three weeks	Anode: central prefrontal area (FPz), cathode: CP6 Cp5.	EEG	The average number of epileptiform	Continuous monitoring of epileptic activity throughout tDCS improves safety and permits detailed evaluation of epileptic activity changes induced by tDCS in patients.
San-Juan et al., 2016	28	tDCS	2 mA, 30 min	3 or 5 sessions in consecutive days	Anode: silent area. cathode: most active interictal epileptiform discharges area	EEG	Seizure frequency	Cathodal tDCS (applied 3 and 5 sessions) reduced seizure frequency and interictal epileptiform discharges for patients with epilepsy and hippocampal sclerosis compared to placebo tDCS.

tDCS remains the primary intervention for epilepsy rehabilitation. Three of the studies applied HD-tDCS as the stimulation protocol with different stimulation parameters and locations. No adverse reactions were reported in any studies, but the reported results differed slightly. One of the studies (Meiron et al., 2019) stated that reduced interictal epileptic discharge and seizure-related delta activity changed. The stimulation intensity was set at 1 mA for 20 min in 20 interventions. Similarly, the interictal sharp wave amplitude after HD-tDCS (0.1–1 mA, 20 min, 10 sessions) was lower than the preintervention level. However, the seizure frequency was not significantly decreased (Meiron et al., 2018). On the other hand, Karvigh et al. (2017) reported that the mean seizure frequency decreased immediately after HD-tDCS (2 mA, 20 min, and 10 sessions). However, this reduction did not persist after 1 month of follow-up. Interestingly, the attention and working memory performance were improved in all patients even after 1 month. These discrepancies in results may have resulted from differences in stimulation protocol parameters (intensity, duration, and location). In five studies, cathodes were placed on the epileptogenic focus (i.e., focal disease-related area) to achieve adequate stimulation (Fregni et al., 2006; Auvichayapat et al., 2013; San-Juan et al., 2017; Lin et al., 2018; Tecchio et al, 2018). Although the stimulation parameters varied, a reduction in seizure frequency or a significant decrease in epileptic discharge was observed. Partial epileptic participants showed reduced seizure frequency when the anode–cathode pair was located between the temporoparietal and contralateral supraorbital regions (Tekturk et al., 2016; Yang et al., 2019a). In one of the studies (Faria et al., 2012), tDCS and EEG recordings were combined to investigate the feasibility of the simultaneous use of two modalities for continuous monitoring of epilepsy. The simultaneous recording signals were used to analyze the reduced interictal epileptiform EEG discharges. The feasibility of this technique may provide an approach for monitoring epileptic activity in real time during the intervention. However, the input current from the stimulation might also interfere with the recording of endogenous EEG signals. Therefore, future studies should consider the advantages and disadvantages of using these two tES modalities simultaneously.

## Schizophrenia

Schizophrenia is a type of psychosis that presents distortions in cognition, thinking, perception, feeling, emotions, and language. Patients experience hallucinations and delusions. For instance, patients may see/hear nonexistent voices/things and develop supernatural beliefs. Quality of life for schizophrenia patients is highly influenced by various risk factors, such as diabetes, cardiovascular disease, and suicide (Patel et al., 2014). The underlying mechanism remains poorly understood, and the gap between research and practical applications is considerable. Further investigation is required to arrive at a better diagnosis and effective therapeutic strategies.

Nine schizophrenia studies were included in this review (Table 6). All the studies employed EEG as the neuroimaging modality. The extracted EEG features consisted of the power spectrum (e.g., delta, theta, and gamma frequency bands), event-related potentials (e.g., P300, P170, etc.), and functional connectivity. The prefrontal cortex plays a crucial role in working memory, cognition, planning, decision making, emotional regulation, and social interaction (Ferguson and Gao, 2018). In over half of the examined studies (five of nine), electrodes were placed on the prefrontal cortex. Only one study (Rassovsky et al., 2018) reported no significant cognitive or neurological (i.e., event-related potentials) effects in patients with schizophrenia. The remaining four studies (Hoy et al., 2015; Dunn et al., 2016; Ahn et al., 2019; Boudewyn et al., 2020) demonstrated improvement following the intervention, either in the behavioral (working memory performance, steady-state auditory response, and proactive cognitive control) or the neurological domains (functional connectivity, gamma oscillation, alpha oscillation, and P300). A comparative study (tDCS, tACS, and sham) (Ahn et al., 2019) reported that 10-Hz tACS stimulation achieved better performance than tDCS in enhancing alpha oscillation modulation of functional connectivity in the alpha band. A similar study (Singh et al., 2019) found that theta oscillations were significantly elevated following theta frequency stimulation, but this phenomenon was not seen for delta frequency with tACS. An improved theta oscillation was also found following medial frontal tDCS in another study (Reinhart et al., 2015), in which tDCS modulated low-frequency oscillation in the absence of synchrony in the patient’s brain. Some research teams have shifted the anodes’ locations from the frontal cortex to the temporal or occipital lobes to probe the effects on the abnormal symptoms of schizophrenia (visual processing abnormalities, working memory deficits, and auditory hallucinations). Working memory performance and auditory deviance detection were increased by anodal frontal and temporal tDCS (Impey et al., 2017). The plasticity effect following occipital tDCS was not found during anodal and cathodal stimulation (Jahshan et al., 2020). Although significant positive effects were not observed in the investigations, a comparison of the results reveals the importance of parameter selection and emphasizes comprehensive research requirements for future studies.

**TABLE 6:** Studies and experimental characteristics of tFS literature for schizonhrenia.

**Authors**	**Subjects**	**Stimulation parameters**				**Neuroim aging**		**Conclusion**
		**Type**	**Current**	**Duration**	**Location**	**Type**	**Feature**	
Boudewyn et al., 2020	37	tDCS	2 mA, 20 min	1 session	Anode: left dorsolateral PFC(F3), Cathode: right supraorbital site (FP2)	EEG	The power spectrum of the gamma band (30–80 Hz)	Gamma oscillations in proactive cognitive control and frontal tDCS may be a promising approach to enhancing proactive cognitive control in schizophrenia.
Jahshan et al., 2020	27	tDCS	2 mA, 20 min	3 sessions for one week	Anode: central occipital cortex, cathode: right shoulder.	EEG	Visual evoked potentials	It is no evidence of an input-specific plasticity effect and an inconsistent effect of tDCS delivered before visual stimulation on plasticity in people with schizophrenia.
Ahn et al., 2019	22	tACS and tDCS	1.5 or 2 mA, 10 Hz, 20 min	10 sessions	Anode: prefrontal cortex (F3, Fp1), cathode: T3, P3	EEG	Alpha oscillations, Power spectral density, functional connectivity.	tACS has potential as a network-level approach to modulate reduced neural oscillations in relation to clinical symptoms in patients with schizophrenia.
Rassovsky et al., 2018	38	tDCS	2 mA, 20 min	Twice daily for three visits	Anode: DLPFC, cathode: right supraorbital	EEG	P300 and N170	There was no significant improvement based on the results of the neurological and cognitive perspective after single-session tDCS.
Dunn et al., 2016	36	tDCS	1 mA, 20 min	1 session	Anode: bilateral DLPFC (FP1 and FP2), cathode:^ right upper arm	EEG	P300	tDCS can engage and modulate an EEG-based auditory processing measure in schizophrenia.
Hoy et al., 2015	16	tDCS	1 or 2 mA, 20 min	3 sessions			Gamma event-related synchronization and correlation.	tDCS may enhance working memory in schizophrenia by restoring normal gamma oscillatory function
					Anode: frontal cortex (F3), cathode: right supraorbital	EEG		
Smith et al., 2015	19	tDCS	1.5 mA, 20 min	1 session	Anode: medial frontal cortex	EEG	Interregional phase synchrony, event-related potential. Power spectrum	Behavioral performance improved. These results provide unique causal evidence for theories of executive control and cortical dysconnectivity in schizophrenia.
Singh et al., 2019	9	tACS	1 mA, 20 min	1 session	Anode: cerebellar vermis, cathode: right shoulder	EEG	Mean relative power at delta (1–4 Hz) and theta (4–8 Hz) frequency bands	Theta oscillations were obtained. Single-session theta frequency stimulation may modulate task-related oscillatory activity in the frontal cortex.
Impey et al., 2017	12	tDCS	2 mA, 20 min	2 sessions	Anode: left auditory or frontal cortex, cathode: contralateral forehead.	EEG	Event-related potential	Anodal frontal tDCS significantly increased working memory performance, which positively correlates with mismatch negativity-tDCS effects.

## Parkinson’s Disease

PD is a neurodegenerative disorder that predominantly impacts the dopamine-producing neurons of the substantia nigra in the brain and further affects movement control. PD patients may experience outward symptoms such as tremors, bradykinesia, limb rigidity, and gait/balance problems. Treatment strategies are dependent on symptoms and may include medication therapy, surgical therapy, and lifestyle modification. Recently, deep brain stimulation (DBS) was approved by the Food and Drug Administration (FDA) to rehabilitate PD patients. However, the procedure for setting the DBS device requires a surgical step. Therefore, researchers have moved to the NIBS area, which seeks noninvasive therapy to avoid surgical pain.

Three PD studies (see Table 7) were included after screening by related keywords. Two of three studies applied EEG to describe the cortical activity, and one employed fNIRS as the neuroimaging modality. The extracted EEG/fNIRS-based features and applied stimulation protocols are varied. One of the studies (Schoellmann et al., 2019) used tDCS to deliver a current of 1 mA from the left sensorimotor cortex for 20 min and the return electrode to the right frontal lobe. After the intervention, the motor cortex’s cortical activity and synchronization were altered, but it did not change the cortical oscillatory of PD patients. However, the reduced beta rhythm oscillation was obtained by the theta-tACS and tRNS, in which research was performed in a personalized stimulation setting (Del Felice et al., 2019). One fNIRS research (Beretta et al., 2020) examined the tDCS effect with different intensities (1 mA, 2 mA, and sham). Both tDCS intensities showed a reduced time response to recover the balance post perturbation. The 2-mA stimulation displayed a better performance than that of 1 mA.

**TABLE 7:** Studies and experimental characteristics of tES literature for PD.

**Authors**	**Subjects**	**Stimulation parameters**				**Neuroimaging**		**Conclusion**
		**Type**	**Current**	**Duration**	**Location**	**Type**	**Feature**	
Del Felice et al., 2019	15	tACS++tRNS	4 or 30 Hz, 1 to 2 mA, 30 min	5 sessions per week for two weeks	Anode: The location of power spectral difference was detected, cathode: ipsilateral mastoid	EEG	Power spectral in delta (1–4 Hz), theta band (4.5–7.5 Hz), alpha1 (8–10 Hz), alpha2 (10.5–12.5 Hz), beta (13—0 Hz)	Individualized tACS in PD improves motor and cognitive performance. These changes are associated with a reduction of excessive fast EEG oscillations.
Beretta et al., 2020	24	tDCS	1 and 2 mA, 20 min	3 sessions 2 weeks apart	Anode: primary motor cortex, cathode: contralateral supraorbital region	fNIRS	The concentration changes of HbO	tDCS over M1 improved the postural response to external perturbation in PD, with better response observed for 2 mA compared with 1 mA, and was inefficient in modifying the habituation of perturbation
Schoellmann et al., 2019	21	tDCS	1 mA, 20 min	1 session	Anode: left sensorimotor (C3), cathode: right frontal area (FP2).	EEG	Frequency domain spectrum and coherence	tDCS improved PD motor symptoms. Neurophysiological features indicated amotor-task-specific modulation of activityand coherence from 22 to 27 Hz after ‘verum’stimulation in PD.

## Stroke

Stroke, known as cerebrovascular disease, is the second leading cause of death and the third leading cause of disability worldwide. The risk of death depends on the category of stroke (transient ischemic attack, blockage of an artery, carotid stenosis, and rupture of a cerebral blood vessel). Since brain cells die due to insufficient blood support, survivors typically experience paralysis, vision or speech loss, and confusion. Several behavioral rehabilitation approaches, such as physical therapy and speech training, are provided to recover impaired brain regions for these patients. NIBS is an emerging method for facilitating neural plasticity in the damaged brain from a neurological perspective, and its potential and capability have received much attention.

Most of the studies (12 of 14) used EEG to monitor the effects of stimulation, as shown in Table 8. Additionally, two studies investigated damaged neurovascular coupling in stroke survivors using a combination of EEG and fNIRS modalities. Both joint EEG-fNIRS studies (Dutta et al., 2015; Jindal et al., 2015) concluded that anodal tDCS could modulate impaired neurovascular coupling. In particular, the log-transformed EEG mean power within 0.5–11.25 Hz correlated with the hemodynamic response of HbO (initial dip). Moreover, functional connectivity (Nicolo et al., 2018), event-related potentials (D’Agata et al., 2016), event-related desynchronization (Kasashima et al., 2012; Ang et al., 2015; Kasashima-Shindo et al., 2015; Naros and Gharabaghi, 2017), and power spectra (theta, alpha, beta, and gamma EEG bands) (Hordacre et al., 2018; Bao et al., 2019; Mane et al., 2019) were also possible biomarkers for checking recovery after tDCS. In the EEG studies, three studies explored the feasibility of tDCS for treating apraxia (swallowing apraxia and aphasia) in stroke. Lower current intensities (1 and 1.2 mA) were used over long-term (10 and 15 sessions, respectively) interventions. Positive behavioral and neurological results were obtained after tDCS for all three studies (Dominguez et al., 2014; Wu et al., 2015; Yuan et al., 2017). Anodal tDCS may offer a novel therapeutic means for the rehabilitation of aphasia and swallowing apraxia. Although all 14 stroke-related investigations applied different stimulation strategies, encouraging postintervention effects were achieved from either the neurological or cognitive perspective. These findings differ from the experimental results for other diseases (e.g., AD or schizophrenia), in which the various stimulation sites and parameters may lead to contrary results. Future studies should explore the underlying altered mechanism by monitoring prognostic neurological or hemodynamic biomarkers. Moreover, attention should be devoted to investigating HbO and neurovascular coupling concentration changes before and after the intervention, especially for small vessel diseases (such as stroke).

**TABLE 8:** Studies and experimental characteristics of tES literature for stroke.

**Authors**	**Subjects**	**Stimulation parameters**				**Neuroim aging**		**Conclusion**
		**Type**	**Current**	**Duration**	**Location**	**Type**	**Feature**	
Mane et al., 2019	19	tDCS	1 mA, 20 min	10 sessions	Anode: ipsilesional primary motor cortex, cathode: contralesionally primary motor cortex	EEG	Power spectral in delta (1–4 Hz), theta band (4–7.5 Hz), alpha (7.5–12.5Hz), beta (12.5–30 Hz), and correlation analysis.	QEEG features can act as prognostic and monitory biomarkers. tDCS-BCI can be pursued to predict a patient’s expected response to an intervention uniquely.
Bao et al., 2019	30	HD-tDCS	1 mA, 10 min	4 sessions	Anode: ipsilesional motor cortex(C3), cathode: frontal-parietal cortex (F1, F5, P1, P5)	EEG	Cortico-muscular coherence and power spectral in alpha (8–13 Hz), beta (13–30 Hz), and low gamma (30–48 Hz).	Anode HD-tDCS induced significant CMC changes in stroke subjects. The largest neuromodulation effects were observed at 10 min immediately after anodal HD-tDCS.
Hordacre et al., 2018	10	tDCS	1 mA, 20 min	1 session	Anode: primary motor cortex, cathode: contralateral orbit	EEG	Connectivity in different frequency band delta (1–3 Hz), theta (4–7 Hz), alpha (8–13 Hz), low beta (14–19 Hz), high beta (20–0 Hz), and gamma (31–45 Hz).	Alpha band functional connectivity of an approximate ipsilesional sensorimotor and contralesionally motor-premotor network is a robust and specific biomarker of neuroplastic induction following anodal tDCS in chronic stroke survivors.
Nicolo et al., 2018	41	tDCS	1 mA, 25 min	3 sessions per week for 3 weeks	Anode: ipsilesional supraorbital region, cathode: contralesionally primary motor cortex	EEG	Effective connectivity and functional connectivity	The inhibition of the contralesionally primary motor cortex or the reduction of interhemispheric interactions was not clinically useful in aheterogeneous group of subacute stroke subjects. Enhancement of perilesional beta-band connectivity through tDCS might have more robust clinical gains if it started within the firstfour weeks after the onset of stroke.
Naros and Gharabaghi, 2017	20	tACS	1.1 mA, 20 Hz, 20 min	1 session 5 sessions per week for 3 weeks	Anode: ipsilesional sensorimotor cortex, cathode: contralesionally forehead.	EEG	Ipsilesional and contralesionally beta power in resting state and event related desynchronization	Intermittent β-tACS reduces the instantaneous variance of sensorimotor β oscillations and increases the specificity of brain self-regulation-based neurofeedback in patients with stroke patients.
Yuan et al., 2017	9	tDCS	1.2 mA, 20 min		Anode: primary sensorimotor cortex, cathode: contralateral shoulder	EEG	Approximate entropy	After tDCS, scores of swallowing apraxia assessments increased, and ApEn indices increased in both stimulated and non-stimulated areas.
D’Agata et al., 2016	34	tDCS	1.5 mA, 20 min	10 daily sessions for 2 weeks	Anode: damaged hemisphere corresponding to motor cortex (C3 or C4), cathode: opposite hemisphere	EEG	Event-related potential (P300, N200)	NIBS generally improved ERP, but transitorily. More than one NIBS cycle (2–4 weeks) should be used in rehabilitation to obtain clinically relevant results after a washout period only in responder patients.
Dutta et al., 2015	4	tDCS	0.526 A/m^2^, 15 min	1 session	Anode: motor cortex (Cz), cathode left supraorbital notch	EEG and fNIRS	The concentration changes of HbO and HbR, power spectrum	The initial dip in HbO2 at the beginning of anodal tDCS corresponded with an increase in EEG’s log-transformed mean power within the 0.5 Hz –11.25 Hz frequency band.
Kasashima-Shindo et al., 2015	18	tDCS	1 mA, 10 min	5 days per week for 2 weeks	Anode: primary sensorimotor cortex of the affected hemisphere, cathode: contralateral supraorbital area.	EEG	Event-related desynchronization	Event-related desynchronization was significantly increased in the tDCS- brain-computer interface group; anodal tDCS can be a conditioning tool for brain-computer interface training in patients with severe hemiparetic stroke.
Wu et al., 2015	12	tDCS	1.2 mA, 20 min	5 sessions per week for 4 weeks	Anode: left posterior peri-sylvian region, cathode: unaffected shoulder	EEG	Approximate entropy	A-tDCS over the left PPR coupled with speech-language therapy can improve picture naming and auditory comprehension in aphasic patients. Moreover, tDCS could modulate the related brain network, not only the stimulated brain areas.
Jindal et al., 2015	29	tDCS	0.526 A/m^2^, 3 min	1 session	Anode: motor cortex (Cz), cathode: frontal cortex (F3 or F4)	EEG and fNIRS	The concentration changes of HbO and HbR, power spectrum	Anodal tDCS can perturb local neural and vascular activity, which can be used for assessing the functionality of regional cerebral microvessels where crematory clinical studies are required in small vessel diseases.
Dominguez et al., 2014	1	tDCS	1 mA, 20 min	5 sessions per week for 3 weeks	Anode: left frontal area. cathode: homologous right contra-lateral area	EEG	Coherence and power spectrum in the delta (0–4 Hz), theta (4–8 Hz), alpha (8–13 Hz), and beta (13–30 Hz) bands.	tDCS can be affected for behavioral performance and inhibit the irregular activity in the right hemisphere. A longer stimulus can produce greater recovery.
Kasashima et al., 2012	6	tDCS	1 mA, 10 min	1 session	Anode: primary motor cortex of the affected hemisphere, cathode: opposite side in thesupraorbital region	EEG	Event-related desynchronization	Anodal tDCS can increase mu ERD of the affected hemisphere in patients with severe hemiparetic stroke as well as in healthy persons.
Ang et al., 2015	19	tDCS	1 mA, 20 min	5 sessions per week for 2 weeks	Anode: silent area. cathode: most active interictal epileptiform discharges area	EEG	Seizure frequency and laterality coefficient.	tDCS improved the motor ability assessment score, and EEG laterality coefficients were improved after the intervention.

## Traumatic Brain Injury

TBI is a disorder characterized by disrupted brain function that is generally caused by an external bump, violent blow, or jolt to the head. TBI may cause a wide range of symptoms due to various brain injury regions, including physical impairment, psychological changes, and sensory or cognitive alterations. Like the rehabilitation method for stroke, the most popular rehabilitation therapies for TBI include physical training, cognitive therapy, and psychological counseling. As reported in animal studies, tES can improve motor deficits by changing the neuroplasticity of TBI. NIBS may provide the potential to promote cognitive or motor recovery in TBI patients.

As shown in Table 9, there are four relevant types of research exploring the feasibility of tDCS for TBI treatment, in which EEG was used to examine neurological alterations. Although the studies employed various stimulation strategies, the outcomes, either in terms of behavioral measurement or neuroimaging, showed positive effects. For example, the positive effects included increased excitability of the sensorimotor brain area (Ulam et al., 2015), improvement of consciousness as measured by the revised JFK coma recovery scale (Straudi et al., 2019; Zhang et al., 2020), improved auditory memory function (O’Neil-Pirozzi et al., 2017), and reduced apathy level (Zhang et al., 2020). Most of these studies employed longer-term stimulation (i.e., 3, 10, and 40 sessions) with a higher current intensity (2 mA) and delivery of the current at the prefrontal cortex. Although some of the studies used different stimulation protocols, the stimulation effect was still achieved. The underlying mechanism needs to be investigated through a comprehensive evaluation to shine a light for future work. The extracted features included the approximate entropy, power spectra (delta, theta, alpha, and beta bands), and ERP (P300), selected as the characteristics for decoding brain signals to assess interventions’ effects. Among those EEG features, delta band declines were significantly associated with neuropsychological test performance following tDCS. As the evidence indicates, the EEG pattern may be correlated with the severity of brain injury. Generally, the power in the slow frequency bands (delta and theta) increased, and the high-frequency band’s power was reduced. Therefore, a possible explanation is that reduction in delta band power is a biomarker for recovery from brain injury in TBI and can be considered a reversed neurological symptom. Therefore, reversed features can be biomarkers for the initial diagnosis to monitor the effects of stimulation.

**TABLE 9:** Studies and experimental characteristics of tES literature for TBI.

**Authors**	**Subjects**	**Stimulation parameters**				**Neuroimaging**		**Conclusion**
		**Type**	**Current**	**Duration**	**Location**	**Type**	**Feature**	
Zhang et al., 2020	10	tDCS	2 mA, 20 min	twice daily, 5 sessions per week for 4 weeks	Anode: Prefrontal area and left DLPFC, cathode: neck and F4.	EEG	Approximate entropy and cross-approximate entropy Relative power in	A-tDCS over the prefrontal area and left DLPFC improves psychomotor inhibition state. The recovery might be related to increased excitability in local and distant cortical networks connecting the sensorimotor area to the prefrontal area.
Straudi et al., 2019	10	tDCS	2 mA, 40 min	5 sessions per week for 2 weeks	Anode: bilaterally primary motor cortex, cathode: Nasion.	EEG	delta (1–3.5 Hz), theta (3.5–7.5 Hz), alpha1 (8–10 Hz), alpha2 (11–13 Hz), beta1 (13.5–18 Hz), beta2 (18.5–30 Hz)	This study tested and evaluated the preliminary effects of bilateral anodal transcranial direct current stimulation in patients with disorders of consciousness.
O’Neil-Pirozzi et al., 2017	8	tDCS	2 mA, 20 min	3 sessions (48 h apart)	Anode: left DLPFC, cathode: right supraorbital area.	EEG		Individuals with memory impairments
							Power spectrum in theta (4–8 Hz), alpha	secondary to chronic TBI may benefit from
							(8–13 Hz), and P300.	LDLPFC anodal tDCS.
							Power spectrum in	
Ulam et al., 2015	26	tDCS	1 mA, 20 min	10 sessions consecutive day	Anode: left DLPFC (F3), cathode: right supraorbital area (Fp2).	EEG	delta (1–4 Hz), theta (4–8 Hz), alpha (8–10 Hz), beta1 (12–25 Hz), beta2 (25–30 Hz)	Ten anodal tDCS sessions may beneficially modulate regulation of cortical excitability for patients with TBI.

## Discussion

**FIGURE 4:**
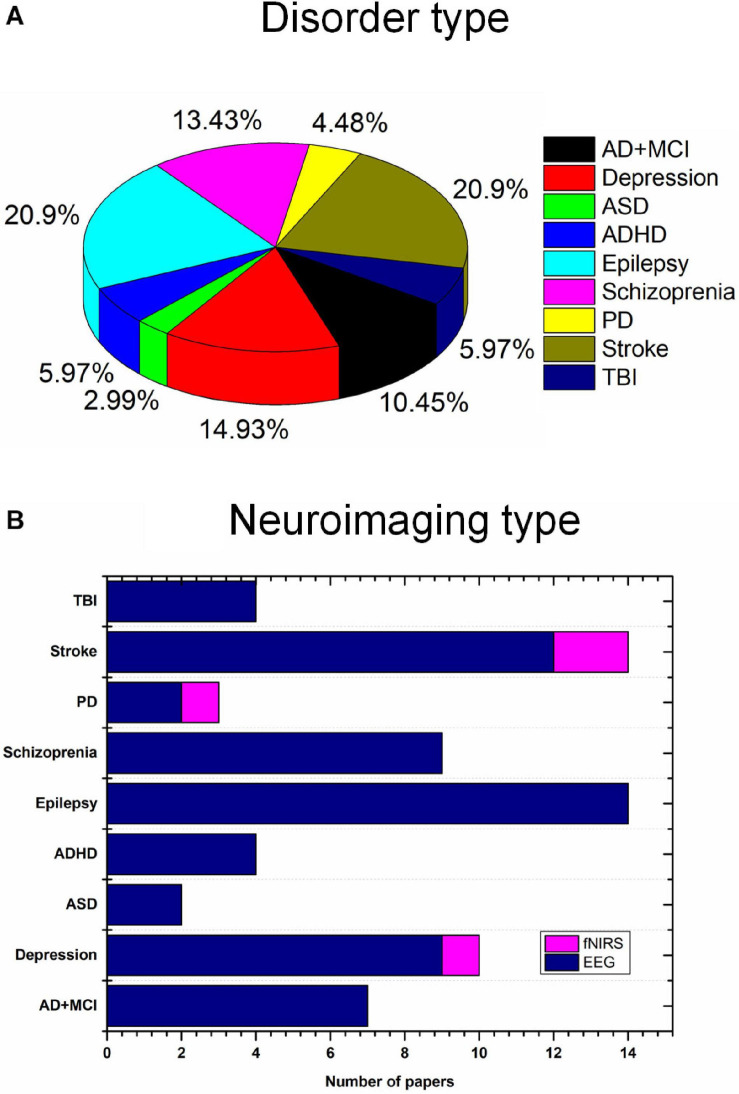
Brain disorders and imaging techniques: **(A)** Nine brain disorders, **(B)** number of EEG/fNIRS papers per brain disorder.

In this study, we investigated the current state of tES utilizing noninvasive neuroimaging techniques (EEG and fNIRS) to monitor altered neurological activity in brain disorders, including AD, MCI, depression, ASD, ADHD, epilepsy, schizophrenia, PD, stroke, and TBI. Moreover, this study presents general guidelines for selecting stimulation parameters for tES and examining the extracted EEG/fNIRS-based features, based on the results of 67 studies with a dataset of 1,385 patients. Epilepsy (20.9%), stroke (20.9%), and depression (14.93%) were most commonly considered. Most of the studies utilized EEG as the neuroimaging technique (Figure 4). This study is the first work to investigate the integration of noninvasive neuroimaging and neuromodulation methods in nine brain disorders to the best of our knowledge.

### Stimulation Modality of tES

**FIGURE 5:**
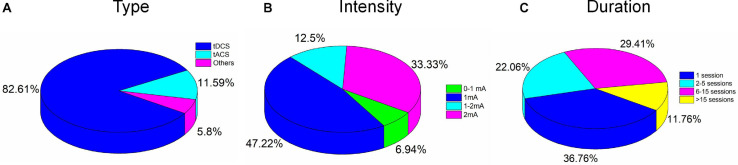
Percentages of electrical stimulation parameters: **(A)** Type, **(B)** intensity, and **(C)** duration.

tDCS is still a representative NIBS, and approximately 82.61% of the investigated studies employed tDCS as the stimulation modality. tACS and the other modalities accounted for 11.59 and 5.8%, respectively, as shown in Figure 5A. The other modalities included slow oscillation between tDCS and tRNS. There is no significant evidence to support the superiority of the treatment effect for each stimulation modality. However, some comparative studies have concluded that tACS or tRNS are the most effective NIBS methods for rehabilitating stroke (Inukai et al., 2016) or schizophrenia (Ahn et al., 2019). These claims still require validation by large sample sizes and multiple examination perspectives. Generally, tDCS cannot modulate the specific frequencies of oscillation but can induce excitability/inhibition, altering certain regions’ cortical activity. Decreasing *r*-aminobutyric acid (GABA) and increasing glutamate/glutamine concentrations have been reported as the physiological mechanisms of tDCS (Reed and Cohen Kadosh, 2018). Declines in GABA have, in turn, been associated with the alteration of resting-state connectivity. It is consistent with studies that have demonstrated changes in functional connectivity following tDCS intervention. Unlike tDCS, tACS can entrain a large number of neuronal firing events with an exogenous frequency. Many studies have stated that tACS can selectively entrain brain oscillations. The entrainment of tACS is most effective when the endogenous oscillation is similar to the frequency of stimulation (Fröhlich and McCormick, 2010). This observation may explain the reported dependence of tACS on brain states. Similarly, altered GABA levels have been observed after tACS intervention (Nowak et al., 2017). Therefore, functional connectivity would be expected to change following stimulation. A future study could consider functional connectivity as an altered biomarker for monitoring the tES effect. The mechanism underlying tRNS, a novel tES modality for stimulating the human brain, is still not clearly understood. One research group (Inukai et al., 2016) concluded that tRNS is more effective than tDCS or tACS for inducing neural excitability. Since stimulation frequency in tRNS can vary, with normal distribution between 0.1 and 640 Hz, some researchers (Campana et al., 2016) have claimed that the opposite effect of cortical excitability would appear after low- and high-frequency stimulation. Two hypotheses have been proposed to explain physiological and pharmacological results. One states that tRNS induces random cortical activity and boosts the brain’s sensitivity to further external input (Van Doren et al., 2014). Alternatively, the other hypothesis holds that repeated random stimulation prevents homeostasis in the brain and potentiates event-related neural activity (Fertonani et al., 2011). Much work remains to identify the mechanism underlying tRNS and obtain a reliable stimulation protocol.

### Stimulation Intensity and Duration of tES

**FIGURE 6:**
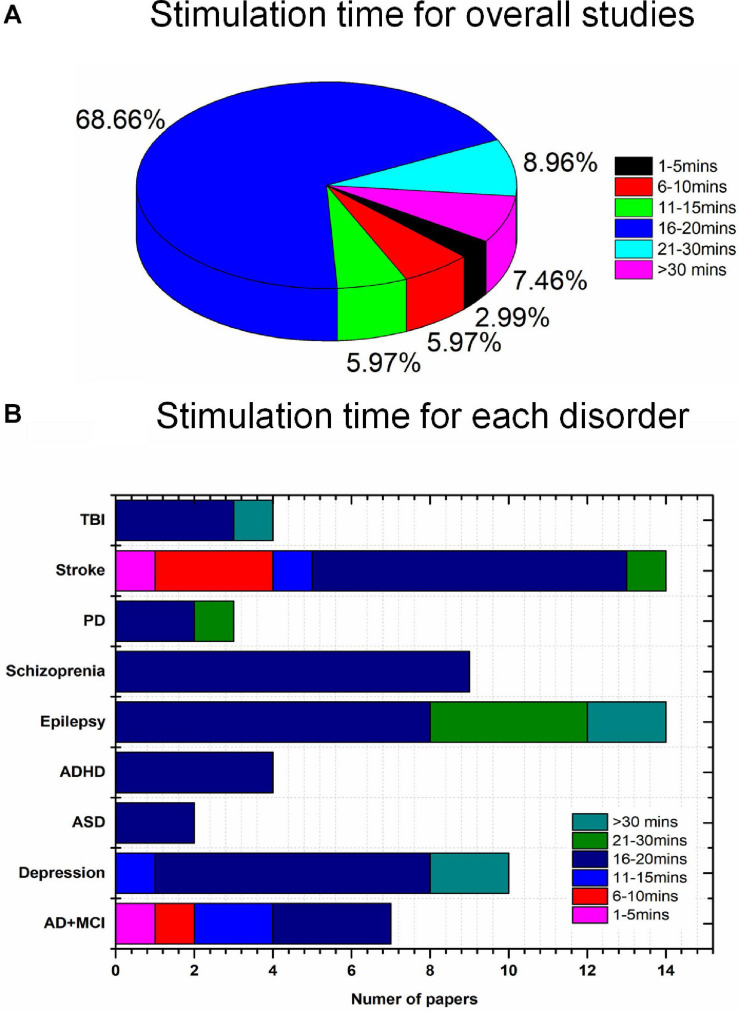
Disease-wise stimulation time distribution (total 67 studies): **(A)** overall, **(B)** disease-wise.

None of the 67 studies reported adverse effects. Conventionally, to avoid skin burns or irritation due to the increased temperature of the electrodes, care must be taken in the choice of stimulation parameters such as duration (<40 min) and intensity (<4 mA) (Bikson et al., 2016). Typically, for tACS and tRNS, patients experience a minimal perception of current relative to tDCS. Overall, differences in duration, intensity, location of stimulation, and combinations of these parameters and tasks performed can result in various postintervention outcomes. Stimulation intensities of 1 mA (47%) and 2 mA (33.3%) were commonly used to deliver the current in the reviewed studies, as shown in Figure 5B. For pediatric participants, the application of reduced current intensities (approximately 1 mA) was suggested. However, a review article with a dataset of 2,800 sessions across approximately 500 pediatric subjects reported that trials with 2 mA did not show severe adverse effects (Bikson et al., 2016). It is consistent with results for studies of children (e.g., ASD, ADHD) included in our review (Tables 3, 4). There was no significant correlation between the stimulation effect and intervention duration. Most of the studies employed a 16–20-min (68.66%) or 21–30-min (8.96%) stimulation for each session, as shown in Figure 6. Although some of the studies applied the intervention in small time windows (<5 min), behavioral or neurological alterations could be observed. Few studies have explored and discussed the appropriate stimulation duration, either long term or short term. Most of the studies conducted tES in a single session (36.76%) or two to five sessions (22.06%), as illustrated in Figure 5C. One of the stroke studies (Dominguez et al., 2014) stated that more prolonged stimuli might lead to more remarkable recovery, which was not consistent with results comparing patients with AD between short- and long-term neurostimulation. In this comparative research, long-term intervention (8 months) slowed AD progression similar to the effect of the short-term intervention (10 days). It is essential to investigate whether it is necessary to apply a long-term neuromodulation session to treat each disorder. Perhaps, a closed-loop stimulation system could overcome this issue, in which the current (e.g., intensity, duration, etc.) is applied based on the real-time brain state.

### Selection of Location for tES Stimulation

**FIGURE 7:**
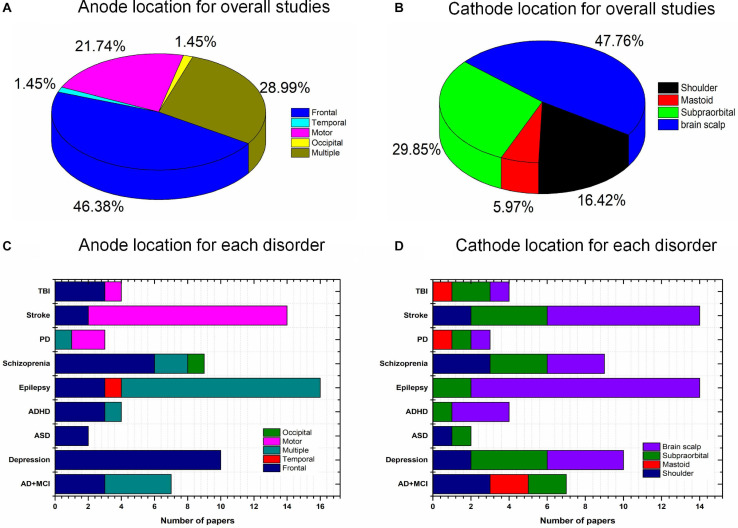
Anode and cathode distributions: **(A)** Anode (overall), **(B)** cathode (overall), **(C)** anode locations for individual diseases, and **(D)** cathode locations for individual disorders.

The placement of the anode and cathode is a crucial factor affecting the stimulation effect. Various intervention results have been achieved owing to the different stimulation locations. The frontal cortex (46.38%) has been widely used for the placement of the anode; in particular, over 80% of studies (Figure 7) of mental disorders (depression, ASD, ADHD, and schizophrenia) delivered current to the frontal brain region. This choice for anode location was mostly due to the specific cortical function and monitored deficits/changes in certain brain areas. For instance, the frontal cortex is used as the region of interest for mental disorders because of its importance in cognition, planning, emotional expression, decision making, and social behavior. Similarly, approximately 85% of stroke studies employed the motor cortex for stimulation due to the symptoms (i.e., trouble walking and loss of balance) in stroke patients. In studies of epilepsy, anodes are positioned in the epileptogenic focus area. Cathodes are usually placed on the supraorbital, mastoid, left/right shoulder, or brain scalp. To date, there is insufficient evidence to support the selection of cathodes to induce particular stimulation effects. Conceptually, the differences in the placement of the return electrode could result in various current trajectories. It would be interesting to investigate the proper placement of the anode and cathode for each brain disorder.

### Neuroimaging in tES

Neuroimaging techniques enable monitoring brain states concerning localized neural activity and the cortical brain network, either offline or online. Neuroimaging is widely used as a monitoring/quantitative tool during or after neuromodulation to elucidate the mechanisms underlying altered symptoms (behavioral and cognitive domains) of brain disorders. It is employed to establish which stimulation protocols respond favorably in terms of symptoms and how the brain is affected from the functional and structural perspectives. Besides, the combination of neuromodulation and neuroimaging techniques enables a causal evaluation of the dynamic interactions among different brain regions (Shafi et al., 2012). As shown in Figure 4B, EEG was used in most tES studies as the neuroimaging method. A few studies utilized fNIRS to measure hemodynamic activity after the intervention. The extracted EEG/fNIRS-based features, such as power spectra, cortical oscillations, network complexity, and functional connectivity, could provide evidence for neuro-modulatory effects. The power spectrum can describe an EEG signal’s power distribution by frequency and time (Kim S. Y. et al., 2020). The commonly applied algorithm is a short-time Fourier transform and wavelet transform. Brain network complexity and connectivity reflect the information transmission status in a brain, determined by entropy analysis, Pearson correlation coefficients, spectral coherence, phase-locking values, and the phase lag index (Song et al., 2019; Fan et al., 2020). Nevertheless, EEG is limited in simultaneous use with the brain stimulation device because recorded signals affect the electric and magnetic fields. However, specific signal-processing algorithms can remove interference components, such as principal component analysis, independent component analysis, and adaptive filter algorithms (Deng et al., 2019; Hong and Pham, 2019; Yoo, 2019; Park et al., 2020). Thus, a technique free of limitation, such as a noninvasive neuroimaging modality (i.e., fNIRS), as a promising method, should be considered the first choice. In addition, hybrid neuroimaging modalities have been suggested to evaluate the neuromodulation effect to seek reliable results (Kwon et al., 2020; Lee S. et al., 2020). For instance, hybrid neuroimaging modalities could improve detection accuracy and provide more compelling support from different perspectives (e.g., electrophysiological, electromagnetic, and hemodynamic) to examine cerebral reactions (Duan et al., 2019; Zhang et al., 2019; Tortora et al., 2020). Meanwhile, the neurovascular coupling can be further investigated as a biomarker for specific blood vascular diseases (e.g., stroke).

**FIGURE 8:**
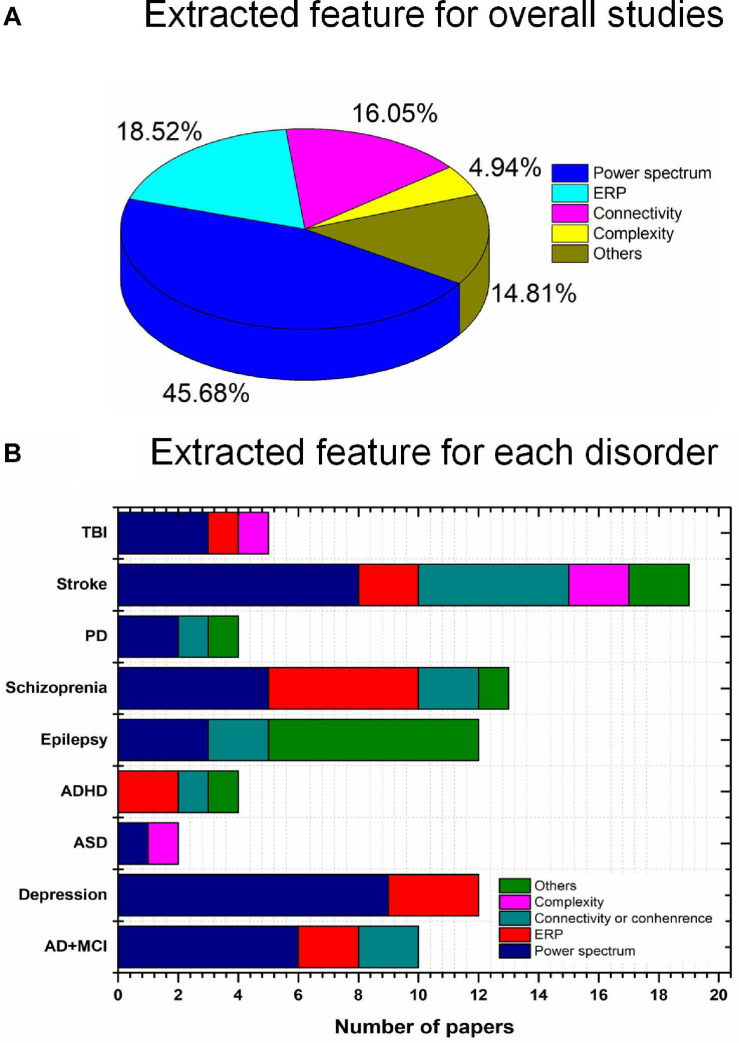
Extracted features (total 67 studies): **(A)** overall and **(B)** individual disorders.

Various EEG- and fNIRS-based features were selected to quantify brain state alterations, including the power spectrum, ERP, brain connectivity, and complexity index. The power spectrum (45.68%) was widely used as the monitoring index to examine modulations’ effects (Figure 8). Most of the studies did not explain feature selection or the mechanisms underlying altered features. Interestingly, one of the tACS studies (Del Felice et al., 2019) analyzed resting-state EEG signals in patients with PD to determine the neural oscillation deficit and brain location affected in each individual. The tACS delivered a sinusoidal fluctuating current with no frequency band to the detected location of the deficit. This investigation may offer a hint that the quantitative features for examining the effects of tES should be extracted based on diagnostic biomarkers in future studies. In other words, the diagnostic biomarker indicated abnormal features of patients relative to healthy controls. Similarly, therapy rehabilitation, in turn, should minimize this difference (diagnostic biomarkers).

### Future Directions

**FIGURE 9:**
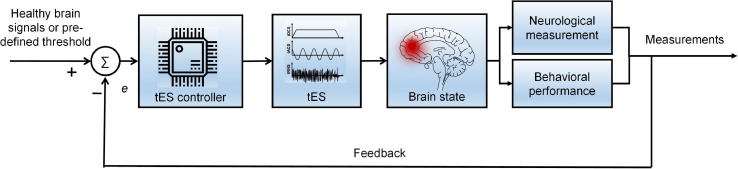
Diagram of the proposed closed-loop tES strategy.

As discussed above, brain states vary among individuals, and standard stimulation criteria are lacking. Therefore, a closed-loop brain stimulation strategy was proposed, providing a customized spatial, temporal, and parameter-specific stimulation protocol for participants based on integrating neuroimaging and neuromodulation techniques. In a strict sense, the closed-loop tES system interactively controls specific variables (current intensity, oscillation frequency, and stimulation duration) using an algorithm to adjust or minimize the error (Anna Paula et al., 2020). Error is generated between the feedback signals and the reference signals (healthy control signals and predefined thresholds) (Lv et al., 2021). Independent variable(s) can be established as any stimulation parameter(s) that need to be optimized (Xu H. et al., 2019). The overall diagram of the closed-loop stimulation is shown in Figure 9. Neurological information (power spectrum and hemodynamic response) and behavioral performance (working memory score and the response time for attention) could quantify the brain state as feedback signals to adjust the system. Future studies should develop a closed-loop tES system by combining different neuroimaging modalities to increase the therapy’s precision and effectiveness. A meta-analysis of a specific disorder/neuroimaging modality/stimulation parameter is also recommended and could reference future studies in quantitative aspects.

## Conclusion

This study investigated 67 studies with a total sample size of 1,385 patients to review the current state of brain disorder therapy applying tES intervention and monitored by noninvasive neuroimaging methods (EEG and fNIRS). Nine brain disorders were reviewed in this article, including AD, depression, ASD, ADHD, epilepsy, schizophrenia, PD, stroke, and TBI. This study presents a conclusive overview of the current development of tES as a neuromodulation modality for the disorders mentioned above. In addition, the summarized stimulation protocols from the 67 studies provide a reference for selecting stimulation parameters for future investigations. Moreover, a closed-loop stimulation strategy was suggested to be a customized tES therapy for patients with brain disease to achieve optimal efficacy and specificity.

## Author Contributions

DY conducted the literature review and wrote the first draft of the manuscript. Y-IS participated in revising the manuscript. K-SH conceived the idea, corrected the manuscript, and finalized the work. All authors have approved the final manuscript.

## Conflict of Interest

The authors declare that the research was conducted in the absence of any commercial or financial relationships that could be construed as a potential conflict of interest.
